# Genetic and Infectious Profiles of Japanese Multiple Sclerosis Patients

**DOI:** 10.1371/journal.pone.0048592

**Published:** 2012-11-09

**Authors:** Satoshi Yoshimura, Noriko Isobe, Tomomi Yonekawa, Takuya Matsushita, Katsuhisa Masaki, Shinya Sato, Yuji Kawano, Ken Yamamoto, Jun-ichi Kira, the South Japan Multiple Sclerosis Genetics Consortium

**Affiliations:** 1 Department of Neurology, Neurological Institute, Graduate School of Medical Sciences, Kyushu University, Fukuoka, Japan; 2 Department of Clinical Neuroimmunology, Neurological Institute, Graduate School of Medical Sciences, Kyushu University, Fukuoka, Japan; 3 Department of Molecular and Genetic Medicine, Ehime University Graduate School of Medicine, Ehime, Japan; 4 Division of Genome Analysis, Research Center for Genetic Information, Medical Institute of Bioregulation, Kyushu University, Fukuoka, Japan; University Hospital La Paz, Spain

## Abstract

**Background:**

Nationwide surveys conducted in Japan over the past thirty years have revealed a four-fold increase in the estimated number of multiple sclerosis (MS) patients, a decrease in the age at onset, and successive increases in patients with conventional MS, which shows an involvement of multiple sites in the central nervous system, including the cerebrum and cerebellum. We aimed to clarify whether genetic and infectious backgrounds correlate to distinct disease phenotypes of MS in Japanese patients.

**Methodology/Principal Findings:**

We analyzed *HLA-DRB1* and *-DPB1* alleles, and IgG antibodies specific for *Helicobacter pylori*, *Chlamydia pneumoniae*, varicella zoster virus, and Epstein-Barr virus nuclear antigen (EBNA) in 145 MS patients and 367 healthy controls (HCs). Frequencies of DRB1*0405 and DPB1*0301 were significantly higher, and DRB1*0901 and DPB1*0401 significantly lower, in MS patients as compared with HCs. MS patients with DRB1*0405 had a significantly earlier age of onset and lower Progression Index than patients without this allele. The proportion and absolute number of patients with DRB1*0405 successively increased with advancing year of birth. In MS patients without DRB1*0405, the frequency of the DRB1*1501 allele was significantly higher, while the DRB1*0901 allele was significantly lower, compared with HCs. Furthermore, DRB1*0405-negative MS patients were significantly more likely to be positive for EBNA antibodies compared with HCs.

**Conclusions:**

Our study suggests that MS patients harboring DRB1*0405, a genetic risk factor for MS in the Japanese population, have a younger age at onset and a relatively benign disease course, while DRB1*0405-negative MS patients have features similar to Western-type MS in terms of association with Epstein-Barr virus infection and DRB1*1501. The recent increase of MS in young Japanese people may be caused, in part, by an increase in DRB1*0405-positive MS patients.

## Introduction

Multiple sclerosis (MS) is an inflammatory demyelinating disease of the central nervous system (CNS). MS is rare in Asians, and is characterized by selective and severe involvement of the optic nerve and spinal cord: this form is termed opticospinal MS (OSMS) [1]. Neuromyelitis optica (NMO) is an inflammatory disease of the CNS selectively affecting the optic nerves and spinal cord. In NMO, longitudinally extensive spinal cord lesions (LESCLs) extending over three or more vertebral segments are regarded as characteristic [2]. The nosological position of NMO has long been a matter of debate. However, the identification of immunoglobulins in NMO patients (NMO-IgG) that are specific for aquaporin-4 (AQP4) indicates that NMO is a distinct disease entity from MS [3], [4]. The classification of NMO has recently been expanded and the limited form of NMO is now named NMO spectrum disorder (NMOSD) [5]. The NMO-IgG/AQP4 antibody is present in 30 to 60% of Japanese OSMS patients [6]–[8]; therefore, OSMS may be a similar entity to NMO. However, more than half of Asian OSMS patients do not have LESCLs, [9] and LESCLs are present in approximately one-fourth of patients with conventional MS (CMS), involving multiple sites of the CNS, including the cerebrum and cerebellum [10], [11]. Thus, in Asians, there is a considerable overlap between MS and NMO. Furthermore, the fourth nationwide survey of MS in Japanese people revealed that the most common type of MS was that with neither Barkhof brain lesions nor LESCLs [9].

MS risk and phenotype may be influenced by multiple genetic and environmental factors [12]. Four nationwide surveys of MS conducted in Japan over thirty years revealed a four-fold increase in the estimated number of clinically definite MS patients in 2003 compared with 1972. In addition, a shift in the peak age at disease onset (from the early 30s in 1989 to the early 20s in 2003) and a successive increase in patients with CMS were observed [13]. This significant increase may be caused by ill-defined environmental changes such as “Westernization”, while reasons for the “earlier age at onset” are unknown.

**Table 1:** Frequencies of *HLA-DRB1* alleles among MS patients and healthy controls.

	Chi-square test or Fisher’s exact probability test			Logistic regression analysis								
	Phenotype frequency			Non adjusted			Adjusted with DRB1*0901			Adjusted with DRB1*0901/DRB1*0405		
	MS (n = 145)	HCs (n = 367)										
DRB1*X	n (%)	n (%)	p^corr^	OR	95%CI	p^corr^	OR	95%CI	p^corr^	OR	95%CI	p^corr^
0101	15 (10.3)	51 (13.9)	1	0.715	0.388–1.317	1	0.584	0.315–1.084	1	0.686	0.365–1.290	1
0403	9 (6.2)	18 (4.9)	1	1.283	0.563–2.926	1	1.239	0.535–2.869	1	1.385	0.591–3.244	1
0405	65 (44.8)	98 (26.7)	0.0013	2.230	1.494–3.330	0.0016	1.939	1.288–2.920	0.0273	NA	NA	NA
0406	17 (11.7)	23 (6.3)	0.6876	1.986	1.028–3.839	1	1.712	0.878–3.337	1	1.917	0.9715–3.783	1
0802	14 (9.7)	26 (7.1)	1	1.402	0.710–2.767	1	1.231	0.618–2.452	1	1.448	0.717–2.923	1
0803	19 (13.0)	58 (15.8)	1	0.803	0.460–1.404	1	0.678	0.385–1.195	1	0.745	0.420–1.323	1
0901	14 (9.7)	101 (27.5)	0.0002	0.282	0.155–0.511	0.0006	NA	NA	NA	NA	NA	NA
1101	5 (3.5)	16 (4.4)	1	0.784	0.282–2.180	1	0.749	0.265–2.114	1	0.822	0.288–2.343	1
1201	12 (8.3)	33 (9.0)	1	0.913	0.458–1.822	1	0.783	0.389–1.573	1	0.922	0.453–1.877	1
1202	2 (1.4)	13 (3.5)	1	0.381	0.085–1.709	1	0.340	0.075–1.539	1	0.349	0.076–1.597	1
1302	8 (5.5)	49 (13.4)	0.1998	0.379	0.175–0.822	0.2516	0.336	0.154–0.733	0.110	0.387	0.176–0.852	0.3300
1403	7 (4.8)	8 (2.2)	1	2.276	0.810–6.396	1	1.961	0.690–1.264	1	2.016	0.700–5.812	1
1405	4 (2.8)	14 (3.8)	1	0.715	0.232–2.210	1	0.666	0.213–2.085	1	0.713	0.225–2.257	1
1406	4 (2.8)	8 (2.2)	1	1.273	0.377–4.295	1	1.206	0.350–4.152	1	1.365	0.391–4.762	1
1454	5 (3.5)	19 (5.2)	1	0.654	0.240–1.786	1	0.607	0.220–1.677	1	0.679	0.244–1.894	1
1501	38 (26.2)	60 (16.4)	0.1908	1.817	1.145–2.885	0.2034	1.627	1.016–2.603	0.767	1.802	1.113–2.916	0.2992
1502	21 (14.5)	80 (21.8)	1	0.608	0.360–1.027	1	0.615	0.361–1.048	1	0.706	0.409–1.217	1
X^a^	13 (9.0)	22 (6.0)										

p^uncorr^ was corrected by multiplying the value by 18 to calculate p^corr^.

X^a^ includes all observed alleles at the *HLA-DRB1* locus with frequencies of less than 1% in subjects; DRB1*0301, DRB1*0401, DRB1*0404, DRB1*0407, DRB1*0410, DRB1*0701, DRB1*1001, DRB1*1106, DRB1*1301, DRB1*1601 and DRB1*1602.

CI, confidence interval; HCs, healthy controls; MS, multiple sclerosis; NA, not applicable; OR, odds ratio; p^corr^, corrected p value.

Sanitary conditions have drastically improved because of the rapid lifestyle Westernization in Japan. Among many potential environmental risk factors, infection is likely to play a significant role in the acquisition of MS susceptibility or resistance. One candidate infectious agent is Epstein-Barr virus (EBV), which is more prevalent in Caucasian MS patients than in healthy controls (HCs) and is, therefore, assumed to increase susceptibility to MS [14], [15]. However, epidemiological surveys suggest that frequent childhood infections may decrease susceptibility to MS [16], [17], as explained by the “hygiene hypothesis” [18]. In Japanese patients, an association of EBV with MS has not yet been demonstrated.

**Table 2:** Frequencies of *HLA-DPB1* alleles among MS patients and healthy controls.

	Chi-square test or Fisher’s exact probability test			Logistic regression analysis								
	Phenotype frequency			non adjusted			Adjusted with DPB1*0301			Adjusted with DPB1*0301/DPB1*0401		
	MS (n = 145)	HCs (n = 367)										
DPB1*X	n (%)	n (%)	p^corr^	OR	95%CI	p^corr^	OR	95%CI	p^corr^	OR	95%CI	p^corr^
0201	58 (40.0)	117 (31.9)	0.8094	1.425	0.957–2.121	0.8161	1.495	0.996–2.242	0.5217	1.385	0.919–2.086	1
0202	8 (5.5)	19 (5.2)	1	1.070	0.457–2.501	1	1.096	0.451–2.533	1	0.965	0.407–2.290	1
0301	21 (14.5)	16 (4.4)	0.0007	3.715	1.879–7.347	0.0016	NA	NA	NA	NA	NA	NA
0401	5 (3.5)	46 (12.5)	0.0198	0.249	0.097–0.641	0.0392	0.257	0.099–0.666	0.0512	NA	NA	NA
0402	27 (18.6)	64 (17.4)	1	1.083	0.659–1.782	1	1.195	0.7217–1.979	1	1.146	0.689–1.905	1
0501	102 (70.3)	240 (65.4)	1	1.255	0.830–1.903	1	1.526	0.981–2.374	0.610	1.351	0.861–2.119	1
0901	23 (15.9)	80 (21.8)	1	0.676	0.406–1.126	1	0.715	0.427–1.197	1	0.706	0.420–1.187	1
1301	4 (2.8)	19 (5.2)	1	0.520	0.174–1.554	1	0.583	0.194–1.747	1	0.594	0.196–1.795	1
1401	4 (2.8)	10 (2.7)	1	1.013	0.313–3.282	1	0.905	0.270–3.036	1	0.877	0.259	1
X^b^	4 (2.8)	5 (1.4)										

p^uncorr^ was corrected by multiplying the value by 10 to calculate p^corr^.

X^b^ includes all observed alleles at the *HLA-DPB1* locus with frequencies of less than 1% in subjects; DPB1*0601, DPB1*1601, DPB1*1701, DPB1*1901, DPB1*2201 and DPB1*4101.

CI, confidence interval; HCs, healthy controls; MS, multiple sclerosis; NA, not applicable; OR, odds ratio; p^corr^, corrected p value.

The largest genetic effect on MS susceptibility is caused by the major histocompatibility complex class II genes. In Caucasians, the HLA-DRB1*1501 allele is strongly associated with MS [19]. Recently, it was reported that the class I allele HLA-A*02 is also associated with MS, independently of DRB1*15, and has a protective effect [20]. In the Japanese population, several studies have reported that CMS is associated with HLA-DRB1*1501, while OSMS is associated with HLA-DPB1*0501 [21], [22]; no association was found with any HLA class I alleles [23]. However, most of these studies were performed before the identification of NMO-IgG, and, therefore, inevitably included NMO patients.

**Table 3:** Comparison of phenotype frequencies of *HLA-DRB1* alleles between MS patients and healthy controls in individuals without the HLA-DRB1*0405 allele.

	MS (n = 80)	HCs (n = 269)			
DRB1*X	n (%)	n (%)	OR	95%CI	p^corr^
0101	11 (13.8)	46 (17.1)	0.773	0.380–1.574	1
0403	5 (6.3)	17 (6.3)	0.988	0.353–2.768	1
0406	11 (13.8)	20 (7.4)	1.985	0.908–4.341	1
0802	10 (12.5)	24 (8.9)	1.458	0.666–3.194	1
0803	15 (18.8)	45 (16.7)	1.149	0.602–2.192	1
0901	10 (12.5)	87 (32.3)	0.299	0.147–0.608	0.0086
1101	4 (5.0)	13 (4.8)	1.036	0.328–3.271	1
1201	7 (8.8)	32 (11.9)	0.710	0.301–1.676	1
1202	2 (2.5)	9 (3.4)	0.741	0.157–3.500	1
1302	6 (7.5)	44 (16.4)	0.415	0.170–1.012	0.8007
1403	3 (3.8)	7 (2.6)	1.458	0.368–5.774	1
1405	4 (5.0)	10 (3.7)	1.363	0.416–4.469	1
1406	3 (3.8)	7 (2.6)	1.458	0.368–5.774	1
1454	3 (3.8)	17 (6.3)	0.578	0.165–2.023	1
1501	29 (36.3)	45 (16.7)	2.831	1.622–4.941	0.0030
1502	17 (21.3)	70 (26.0)	0.767	0.421–1.399	1
X^e^	12 (15.0)	20 (7.4)			

p^uncorr^ was corrected by multiplying the value by 17 to calculate p^corr^.

X^e^ includes all observed alleles at the *HLA-DRB1* locus with frequencies of less than 1% in subjects; DRB1*0301, DRB1*0401, DRB1*0404, DRB1*0407, DRB1*0410, DRB1*0701, DRB1*1001, DRB1*1106, DRB1*1301, DRB1*1601 and DRB1*1602.

CI, confidence interval; HCs, healthy controls; MS, multiple sclerosis; OR, odds ratio; p^corr^, corrected p value.

Thus, in the present study, we analyzed the genetic and infectious profiles of patients from the southern part of Japan with MS who did not fulfill the criteria for NMO or NMOSD. We sought to clarify: i) what the genetic and infectious risk factors for Japanese MS are; ii) whether genetically defined MS subtypes show distinct clinical and neuroimaging features; iii) whether a certain subtype of MS is more prevalent in younger Japanese patients; and iv) whether the profile of common infections are distinct or the same in genetically determined subtypes. In the present study, we focused on *HLA-DRB1* and *-DPB1* loci that are associated with Japanese MS but not HLA class I, which have not shown any association with the disease.

## Methods

### Participants

One hundred and forty-five patients who were examined at the Neurology Departments of the University Hospitals of the South Japan MS Genetic Consortium (Co-investigator Appendix) between 1987 and 2010 were enrolled. MS was defined using the 2005 revised McDonald criteria for MS [24]. NMO was defined as cases fulfilling the 2006 revised criteria for NMO [25]. We regarded patients as having an NMOSD when the patients fulfilled either two absolute criteria plus at least one supportive criterion, or one absolute criterion plus more than one supportive criterion from the 2006 NMO criteria [25], primarily because there is considerable overlap between MS and NMO in Asians, as mentioned in the Introduction section. None of the MS patients met the above-mentioned NMO/NMOSD criteria. Patients with primary progressive MS were excluded from the study. Informed consent was obtained from 145 patients and 367 unrelated HCs. We collected demographic data from the patients by retrospective review of their medical records. These data included gender, age of onset, disease duration, Kurtzke’s Expanded Disability Status Scale (EDSS) scores [26], annualized relapse rate, Progression Index (PI) [27], cerebrospinal fluid (CSF) oligoclonal IgG bands (OB; as determined by isoelectric focusing [28]) and IgG index, brain MRI lesions that met the Barkhof criteria for MS [26], and the presence of LESCLs. The ethics committee of each institution approved this study.

### MRI Analysis

All MRI studies were performed using 1.5 T units (Magnetom Vision and Symphony, Siemens Medical Systems, Erlangen, Germany) as previously described [6], [8]. Brain MRI lesions were evaluated according to the Barkhof criteria for MS [29]. Spinal cord lesions extending over three or more vertebral segments in length were considered to be LESCLs.

**Table 4:** Comparison of demographic features and clinical characteristic of MS patients according to the presence or absence of the HLA-DRB1*0405 allele.

	*0405* (+) (n = 65)	*0405* (−) (n = 80)	p^uncorr^	p^corr^
Male: female	22 : 43	29 : 51	0.8615	1
Age at onset (years)^a^	27.22±10.45	34.84±13.76	0.0014	0.0126
Disease duration (years)^a^	12.83±9.68	10.22±7.27	0.1211	1
EDSS score^a^	2.48±2.05	3.49±2.37	0.0078	0.0702
Annualized relapse rate^a^	0.54±0.45	0.70±0.78	0.2290	1
Progression Index^a^	0.33±0.42	0.66±1.38	0.0017	0.0153
OB/increased IgG index^b^	22/42 (52.4%)	37/52 (71.2%)	0.0859	1
Barkhof criteria^c^	31/58 (53.5%)	51/70 (72.9%)	0.0272	0.2448
LESCLs	3/58 (5.2%)	5/71 (7.0%)	0.7295	1

a Values represent the mean ± SD.

b CSF oligoclonal IgG bands (OB) and/or increased IgG index (upper normal limit = 0.658, according to our previous study [21].

c Brain MRI lesions that meet the Barkhof criteria [29].

EDSS, Kurtzke’s Expanded Disability Status Scale; LESCLs, longitudinally extensive spinal cord lesions extending over three or more vertebral segments; MS, multiple sclerosis; OB, oligoclonal IgG bands.

p^uncorr^ was corrected by multiplying the value by nine to calculate p^corr^.

### *HLA-DRB1* and *-DPB1* Genotyping

The genotypes of the *HLA-DRB1* and *-DPB1* alleles from the subjects were determined by hybridization between the products of polymerase chain reaction amplification of the *HLA-DRB1* and -*DPB1* genes and sequence-specific oligonucleotide probes, as described previously [30].

### AQP4 Antibody Assay

The presence of AQP4 antibodies was assayed as described previously [8], using green fluorescent protein (GFP)-AQP4 (M1 isoform) fusion protein-transfected human embryonic kidney (HEK) cells. Serum samples diluted 1∶4 were assayed for the presence of AQP4 antibodies, and repeated at least twice using identical samples, with the examiners blinded to the origin of the specimens. Samples that gave a positive result twice were deemed positive.

### Detection of Anti-*Helicobacter pylori*, Anti-*Chlamydia pneumoniae*, Anti-varicella Zoster Virus, and Anti-Epstein-Barr Virus Nuclear Antigen IgG Antibodies

Serum anti-*Helicobacter pylori* (*H. pylori*), anti-*Chlamydia pneumoniae* (*C. pneumoniae*), anti-varicella zoster virus (VZV), and anti-Epstein-Barr nuclear antigen (EBNA) IgG antibodies were measured using commercial ELISA kits according to the manufacturer’s instructions (Vircell, Granada, Spain), as described previously [31]. The antibody index was determined by dividing the optical density (O.D.) values for target samples by the O.D. values for cut-off control samples and then multiplying by 10. As recommended by the manufacturer, an ELISA test index value was considered positive if higher than 11, equivocal if between 9–11 and negative if less than 9. Samples with equivocal results were retested for confirmation. Samples that were equivocal twice were considered negative.

**Figure 1:**
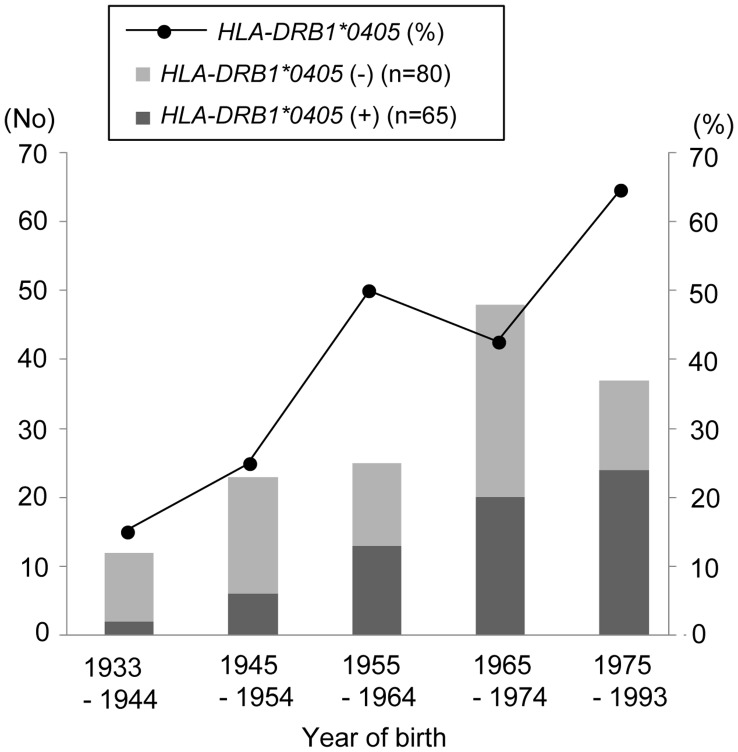
Proportion of HLA-DRB1*0405-positive and -negative MS patients by year of birth. Among MS patients, the proportion and absolute number of patients with DRB1*0405 successively increased with advancing year of birth. MS, multiple sclerosis.

### Statistical Analyses

The phenotype frequencies of the *HLA-DRB1* and *-DPB1* alleles were compared using either the chi-square test or Fisher’s exact probability test (when the criteria for the chi-square test were not fulfilled). We also conducted a dominant model of logistic regression analysis to identify *HLA-DRB1* and *-DPB1* alleles associated with MS for alleles that have frequencies greater than 1% in subjects (cases and controls), and then conditioned on the top associated alleles to identify subsequently associated alleles. Estimation of *HLA-DRB1-DPB1* haplotype frequencies and haplotype-based association analysis were performed using HaploView software. We checked the *HLA-DRB1-DPB1* haplotypes that had frequencies greater than 1% in subjects. We used the Lewontin D’ measure to estimate the intermarker coefficient of linkage disequilibrium in both HCs and MS patients. Uncorrelated p-values (p^uncorr^) were corrected by multiplying them by the number of comparisons, as indicated in the footnote of each table (Bonferroni–Dunn’s correction), to calculate the corrected *p*-values (p^corr^). Fisher’s exact probability test was used to compare gender, CSF IgG abnormalities, brain MRI lesions that met the Barkhof criteria [29], frequencies of antibodies against common infectious agents among subgroups and the presence of LESCLs between subgroups. Other demographic features were analyzed using the Wilcoxon rank sum test. All analyses were performed using PLINK (version 1.07), Haploview (version 4.2), R (version 2.15) and JMP 8.0.3 (SAS Institute, Cary, NC, USA). We used PROC LOGISTIC (SAS Institute) to analyze the trends in the proportions of patients among subgroups with advancing year of birth using the Cochran-Armitage trend test. In all assays, p values <0.05 were considered statistically significant.

**Table 5:** Frequencies of antibodies against common infectious agents among MS patients with and without the HLA-DRB1*0405 allele and healthy controls.

Infections	HCs	HLA-DRB1*0405(−) MS	HLA-DRB1*0405(+) MS
EBV	143/156 (91.67%)	71/71 (100.0%)*a, *b	48/56 (85.71%)
VZV	153/156 (98.08%)	70/71 (98.59%)	56/56 (100.0%)
*Helicobacter pylori*	74/177 (41.81%)	23/71 (32.39%)	21/56 (37.5%)
*Chlamydia*	92/156 (58.97%)	46/71 (64.79%)	32/56 (57.14%)

*a p^corr^ = 0.0324, as compared with HCs.

*b p^corr^ = 0.0033, as compared with HLA-DRB1*0405 (+) MS.

The age of patients during examination was not significantly different among HCs and MS patients, regardless of the presence or absence of the DRB1*0405 allele (mean ± SD in years; 35.22±12.18 in DRB1*0405-positive MS patients, 40.74±12.41 in DRB1*0405-negative MS patients, and 38.93±12.11 in HCs).

EBV, Epstein-Barr virus; HCs, healthy controls; MS, multiple sclerosis; VZV, varicella zoster virus.

## Results

### Frequencies of *HLA-DRB1* and *-DPB1* Alleles

Compared with HCs, the phenotype frequencies of the DRB1*0405 and DPB1*0301 alleles were significantly higher in MS patients (p^corr^ = 0.0013, OR = 2.230, 95% CI = 1.494–3.330, and p^corr^ = 0.0007, OR = 3.715, 95% CI = 1.879–7.347, respectively). By contrast, the frequencies of DRB1*0901 and DPB1*0401 were significantly lower (p^corr^ = 0.0002, OR = 0.281, 95% CI = 0.155–0.511, and p^corr^ = 0.0198, OR = 0.249, 95% CI = 0.097–0.641, respectively) (Tables 1 and 2). Even when a dominant model of logistic regression analysis was conducted to identify associations between *HLA-DRB1* and *-DPB1* alleles and MS for alleles that had frequencies greater than 1% in subjects, we could not find any other associated alleles except for DRB1*0901, DRB1*0405, DPB1*0301, and DPB1*0401. Exclusion of eight MS patients with LESCLs gave essentially the same results (Tables S1 and S2); MS patients showed a higher frequency of DRB1*0405and DPB1*0301, and lower frequency of DRB1*0901 and DPB1*0401 compared with HCs.

### Frequency of *HLA-DRB1* and *-DPB1* Alleles in Subjects Lacking the HLA-DRB1*0405 Allele

Among individuals lacking the DRB1*0405 allele, the frequency of the DRB1*1501 allele was significantly higher (p^corr^ = 0.0030, OR = 2.831, 95% CI = 1.622–4.941) and that of the DRB1*0901 allele was significantly lower (p^corr^ = 0.0086, OR = 0.299, 95% CI = 0.147–0.608) in MS patients compared with HCs (Table 3). The frequency of *DPB1* alleles were not significantly different between MS patients and HCs in subjects without the DRB1*0405 allele (data not shown).

### Frequency of *DRB1-DPB1* Haplotypes

Compared with HCs, the haplotype frequencies in MS patients were significantly increased for the DRB1*0405-DPB1*0301 haplotype (p^uncorr^ = 0.0002, p^corr^ = 0.0042) (Table S3). However, the significance of this association was weaker than that of the DRB1*0405 allele (p^uncorr^ = 7.2×10^−5^, p^corr^ = 0.0013) or the DPB1*0301 allele (p^uncorr^ = 6.7×10^−5^, p^corr^ = 0.0007).

### Comparison of Demographic Features between HLA-DRB1*0405-positive and -negative MS Patients

MS patients positive for DRB1*0405 showed an earlier age of onset, a lower EDSS score, a lower PI and a lower frequency of brain MRI lesions that met the Barkhof criteria [26] compared with patients without this allele (p^uncorr^ = 0.0014, p^uncorr^ = 0.0078, p^uncorr^ = 0.0017, and p^uncorr^ = 0.0272, respectively) (Table 4). The frequency of OB/increased IgG index was also lower in patients with DRB1*0405 than those without the allele, but the difference did not reach statistical significance (p^uncorr^ = 0.0859). Even after Bonferroni–Dunn’s correction for multiple comparisons was made, MS patients positive for DRB1*0405 showed a significantly earlier age of onset and a significantly lower PI compared with those without this allele (p^corr^ = 0.0126 and p^corr^ = 0.0153, respectively). Furthermore, when eight MS patients with LESCLs were excluded, a similar difference in demographic features between MS patients with and without DRB1*0405 were observed (Table S4). Among patients without DRB1*0405, DRB1*1501-positive patients had a significantly higher frequency of CSF OB/increased IgG index than DRB1*1501-negative patients (17/19, 89.5% versus 20/33, 60.6%, p = 0.0312).

### Differences in the Distribution of HLA-DRB1*0405-positive and -negative MS Patients by Year of Birth

The proportion and absolute number of patients with DRB1*0405 successively increased with advancing year of birth (p = 0.0013, and p = 0.0005, respectively) (Figure 1).

### Frequency of Anti-*H. pylori*, Anti-*C. pneumoniae*, Anti-VZV, and EBNA IgG Antibodies in MS and NMO

DRB1*0405-negative MS patients had a higher frequency of EBNA IgG antibodies compared with HCs, and DRB1*0405-positive MS patients (p^corr^ = 0.0324, and p^corr^ = 0.0033, respectively) (Table 5). Anti-*H. pylori*, anti-*C. pneumoniae* and anti-VZV antibody positivity rates did not differ significantly among the three subgroups.

## Discussion

The current study on Japanese MS patients, excluding patients with NMO, identified the following: (1) DRB1*0405 and DPB1*0301 are susceptibility alleles for MS. (2) DRB1*0405-positive MS patients showed an earlier age of disease onset and a relatively benign disease course. (3) The proportion and absolute numbers of DRB1*0405-positive patients among the total MS patients successively increased with advancing year of birth. (4) In DRB1*0405-negative MS patients, DRB1*1501 is a major susceptibility allele. (5) Susceptibility genes vary according to the disease phenotype, while DRB1*0901 is a common protective allele, irrespective of the phenotype. (6) Compared with healthy controls, DRB1*0405-negative MS patients had a significantly higher frequency of EBNA IgG antibodies. In the present study, the effect of the most strongly associated haplotype, the DRB1*0405-DPB1*0301 haplotype, on MS risk was lower than the effect of the DRB1*0405 allele or the DPB1*0301 allele alone. The linkage disequilibrium between the *DRB1* and *DPB1* loci is generally weak in the Japanese population [32]. Therefore, we focused on the association of a single marker, the *DRB1* or *DPB1* allele, which could be more meaningful than *DRB1-DPB1* haplotypes in Japanese MS.

### HLA-DRB1*0405-positive MS

The DRB1*0405 allele was found to be a significant risk determinant among Japanese patients with MS. A subgroup of DRB1*0405-positive MS patients showed distinct features: a younger age at disease onset, lower EDSS scores, a lower PI, and a lower frequency of MS-like brain lesions compared with DRB1*0405-negative patients. In addition, DRB1*0405-positive MS patients demonstrated a tendency for a lower frequency of CSF OB/increased IgG index compared with DRB1*0405-negative MS patients. This is in line with previous findings demonstrating that DRB1*04 is associated with OB-negative MS in Swedish [33] and Japanese populations [34]. A low prevalence of OB (54%) similar to that observed in the present study was also reported in Japanese MS patients as a unique feature compared with Western MS [1], [28]. The MS patients with DRB1*0405 may, in part, be responsible for this low prevalence of OB in Japanese MS patients. Even when Bonferroni–Dunn’s correction was performed, only MS patients positive for DRB1*0405 showed an earlier age of onset and a lower PI compared with patients without this allele. Therefore, DRB1*0405-positive MS could be a unique subgroup of MS that develops with a relatively benign disease course from an earlier age. According to the fourth nationwide survey of MS in Japanese people, the most common type of MS had neither Barkhof brain lesions nor LESCLs [9]. Hence, it is remarkable that such a subtype of MS with DRB1*0405 as a susceptibility risk is the most common in Japanese MS patients while it is present in a relatively minor population of Caucasian MS patients [33]. The proportion and absolute numbers of MS patients with DRB1*0405 have successively increased with advancing year of birth and this group of MS patients has a significantly younger age at disease onset. Therefore, the recently increased numbers of this subgroup of MS patients may explain the recently observed decrease in age at onset in Japanese MS patients, and could be partly responsible for the recent increase of MS prevalence in Japan [13]. However, it is still possible that MS patients with DRB1*0405 and a milder disease might have been previously overlooked and these patients might have been recently diagnosed as having MS owing to the increasingly widespread use of MRI. Thus, our findings should be confirmed by a large-scale study.

### HLA-DRB1*0405-negative MS

DRB1*0405-negative MS patients had higher frequencies of DRB1*1501 and EBNA IgG antibodies compared with HCs. These two factors, which are also identified as risk factors for MS in Caucasians [35], are presumed to be risk factors for MS in DRB1*0405-negative Japanese subjects. DRB1*0405-negative MS patients had a significantly higher frequency of brain lesions fulfilling the Barkhof criteria. In these subjects, the presence of the DRB1*1501 allele was significantly associated with the CSF OB/increased IgG index. These features also resembled those of MS in Westerners [36], [37]. Therefore, this group of Japanese patients represents a “Western” type of MS in terms of clinical, neuroimaging, genetic, and infectious characteristics. The presence of the DRB1*1501 allele promotes the development of more T2 lesions [38] and positively interacts with EBV, a pathogen with a strong correlation to Caucasian MS [19], to increase MS susceptibility and disease burdens [39], [40]. Similar biological mechanisms may occur in Asian patients.

In the current study, DPB1*0301 was shown to be a significant risk allele in MS patients. An association of DPB1*0301 with MS has been reported in residents of Hokkaido in northern Japan [41] and in other ethnically diverse populations, such as Australians [42] and Sardinians [43]. However, the observed clinical features were not significantly different between the DPB1*0301-positive and -negative group in the MS patients from this study (data not shown).

### A Common Genetic Background between HLA-DRB1*0405-positive and -negative MS Patients in Japanese Patients

The current study found that the DRB1*0901 allele had a strong protective effect against MS, regardless of the presence or absence of the HLA-DRB1*0405 allele. A recent meta-analysis in Chinese patients determined that the DRB1*0901 allele was protective for MS [44]. The DRB1*0901 allele is more frequently observed in Asians than in other ethnic groups (Japanese 30% *vs.* Caucasians 1%) [45]. Thus, one explanation for the lower MS prevalence in Japan and other Asian countries may be that the frequency of the DRB1*0901 allele is comparatively higher in those regions.

### Limitations

The present study had some limitations; the numbers of enrolled MS patients were not large because the relative rarity of the disease in the Japanese population. However, this is the largest combined genetic and infection study undertaken in Asian countries, in which well-characterized cases were collected and were processed through the South Japan Multiple Sclerosis Genetics Consortium. In addition, after appropriate corrections for multiple comparisons were made, a number of findings were still statistically significant, which we hope will provide the basis for future studies and which should be confirmed by a large scale study.

## Supporting Information

Table S1 **Frequency of** ***HLA-DRB1*** **alleles among MS patients without LESCLs and healthy controls.** Exclusion of eight MS patients with LESCLs gave essentially the same results; MS patients showed a significantly higher frequency of DRB1*0405, and lower frequency of DRB1*0901 compared with HCs.
(DOCX)

Table S2 **Frequency of** ***HLA-DPB1*** **alleles among MS patients without LESCLs and healthy controls.** Exclusion of eight MS patients with LESCLs gave essentially the same results; MS patients showed a significantly higher frequency of DPB1*0301, and lower frequency of DPB1*0401 compared with HCs.
(DOCX)

Table S3 **Frequency of** ***DRB1-DPB1*** **haplotypes.** Compared with HCs, the haplotype frequencies in MS patients were significantly increased for the DRB1*0405-DPB1*0301 haplotype.
(DOCX)

Table S4 **Demographic features of MS patients without LESCLs according to the presence or absence of the** HLA-DRB1*0405 **allele.** Exclusion of eight MS patients with LESCLs gave essentially the same results; MS patients positive for DRB1*0405 showed a significantly earlier age of onset and a significantly lower PI compared with those without this allele.
(DOC)
