# Supplementary material for: Genetic and Infectious Profiles of Japanese Multiple Sclerosis Patients
Source: PLoS One. 2012 Nov 9;7(11):e48592. doi: 10.1371/journal.pone.0048592 (PMC3494689; doi:10.1371/journal.pone.0048592)
Supplement: Table S1 — Frequency of HLA-DRB1 alleles among MS patients without LESCLs and healthy controls. Exclusion of eight MS patients with LESCLs gave essentially the same results; MS patients showed a significantly higher frequency of DRB1*0405, and lower frequency of DRB1*0901 compared with HCs. (DOCX) [file pone.0048592.s001.docx]

**Supplementary Table 1.** Comparison of phenotype frequencies of *HLA-DRB1* alleles among MS patients without LESCLs and healthy controls

|  | MS (n = 137) | HCs (n = 367) |  |  |  |
| --- | --- | --- | --- | --- | --- |
| *DRB1*X* | n (%) | n (%) | OR | 95%CI | p^corr^ |
| 0101 | 14 (10.2) | 51 (13.9) | 0.705 | 0.377-1.321 | 1 |
| 0403 | 8 (5.8) | 18 (4.9) | 1.202 | 0.510-2.833 | 1 |
| 0405 | 62 (45.3) | 98 (26.7) | 2.269 | 1.509-3.413 | 0.0012 |
| 0406 | 17 (12.4) | 23 (6.3) | 2.119 | 1.095-4.101 | 0.4176 |
| 0802 | 14 (10.2) | 26 (7.1) | 1.493 | 0.755-2.951 | 1 |
| 0803 | 18 (13.1) | 58 (15.8) | 0.806 | 0.456-1.424 | 1 |
| 0901 | 14 (10.2) | 101 (27.5) | 0.300 | 0.165-0.543 | 0.0007 |
| 1101 | 5 (3.7) | 16 (4.4) | 0.831 | 0.298-2.314 | 1 |
| 1201 | 11 (8.0) | 33 (9.0) | 0.884 | 0.433-1.802 | 1 |
| 1202 | 1 (0.7) | 13 (3.5) | 0.200 | 0.026-1.545 | 1 |
| 1302 | 7 (5.1) | 49 (13.4) | 0.349 | 0.154-0.792 | 0.1584 |
| 1403 | 7 (5.1) | 8 (2.2) | 2.416 | 0.859-6.796 | 1 |
| 1405 | 4 (2.9) | 14 (3.8) | 0.758 | 0.245-2.345 | 1 |
| 1406 | 4 (2.9) | 8 (2.2) | 1.350 | 0.400-4.556 | 1 |
| 1454 | 5 (3.7) | 19 (5.2) | 0.694 | 0.254-1.896 | 1 |
| 1501 | 34 (24.8) | 60 (16.4) | 1.689 | 1.049-2.719 | 0.5379 |
| 1502 | 19 (13.9) | 80 (21.8) | 0.578 | 0.335-0.996 | 0.8316 |
| X^c^ | 13 (9.5) | 24 (6.5) |  |  |  |

p^uncorr^ was corrected by multiplying the value by 18 to calculate p^corr^.

X^c^ includes all observed alleles at the *HLA-DRB1* locus with frequencies of less than 1% in subjects; *DRB1*0301, DRB1*0401, DRB1*0404, DRB1*0407, DRB1*0410, DRB1*0701, DRB1*1001, DRB1*1106, DRB1*1301, DRB1*1601* and *DRB1*1602.*

CI, confidence interval; HCs, healthy controls; LESCLs, longitudinally extensive spinal cord lesions extending over three or more vertebral segments; MS, multiple sclerosis; OR, odds ratio; p^corr^, corrected p value.
