# Supplementary material for: Genetic and Infectious Profiles of Japanese Multiple Sclerosis Patients
Source: PLoS One. 2012 Nov 9;7(11):e48592. doi: 10.1371/journal.pone.0048592 (PMC3494689; doi:10.1371/journal.pone.0048592)
Supplement: Table S2 — Frequency of HLA-DPB1 alleles among MS patients without LESCLs and healthy controls. Exclusion of eight MS patients with LESCLs gave essentially the same results; MS patients showed a significantly higher frequency of DPB1*0301, and lower frequency of DPB1*0401 compared with HCs. (DOCX) [file pone.0048592.s002.docx]

**Supplementary Table 2.** Comparison of phenotype frequencies of *HLA-DPB1* alleles among MS patients without LESCLs and healthy controls

|  | MS (n = 137) | HCs (n = 367) |  |  |  |
| --- | --- | --- | --- | --- | --- |
| *DPB1*X* | n (%) | n (%) | OR | 95%CI | p^corr^ |
| 0201 | 56 (40.9) | 117 (31.9) | 1.477 | 0.985-2.215 | 0.5844 |
| 0202 | 8 (5.8) | 19 (5.2) | 1.136 | 0.485-2.659 | 1 |
| 0301 | 19 (13.9) | 16 (4.4) | 3.532 | 1.759-7.092 | 0.0018 |
| 0401 | 5 (3.7) | 46 (12.5) | 0.264 | 0.103-0.680 | 0.0325 |
| 0402 | 25 (18.3) | 64 (17.4) | 1.057 | 0.634-1.761 | 1 |
| 0501 | 96 (70.1) | 240 (65.4) | 1.239 | 0.811-1.894 | 1 |
| 0901 | 22 (16.1) | 80 (21.8) | 0.686 | 0.408-1.153 | 1 |
| 1301 | 4 (2.9) | 19 (5.2) | 0.551 | 0.183-1.649 | 1 |
| 1401 | 3 (2.2) | 10 (2.7) | 0.799 | 0.217-2.949 | 1 |
| X^d^ | 4 (2.9) | 15 (4.1) |  |  |  |

p^uncorr^ was corrected by multiplying the value by 10 to calculate p^corr^.

X^d^ includes all observed alleles at the *HLA-DPB1* locus with frequencies of less than 1% in subjects; *DPB1*0601, DPB1*1601, DPB1*1701, DPB1*1901, DPB1*2201* and *DPB1*4101.*

CI, confidence interval; HCs, healthy controls; LESCLs, longitudinally extensive spinal cord lesions extending over three or more vertebral segments; MS, multiple sclerosis; OR, odds ratio; p^corr^, corrected p value.
