# Supplementary material for: Genetic and Infectious Profiles of Japanese Multiple Sclerosis Patients
Source: PLoS One. 2012 Nov 9;7(11):e48592. doi: 10.1371/journal.pone.0048592 (PMC3494689; doi:10.1371/journal.pone.0048592)
Supplement: Table S3 — Frequency of DRB1-DPB1 haplotypes. Compared with HCs, the haplotype frequencies in MS patients were significantly increased for the DRB1*0405-DPB1*0301 haplotype. (DOCX) [file pone.0048592.s003.docx]

**Supplementary Table 3** Haplotype frequencies of *DRB1*-*DPB1* haplotypes and haplotype associations among MS patients and healthy controls.

|  |  | Frequency (ratio) | | p^uncorr^ | p^corr^ |
| --- | --- | --- | --- | --- | --- |
| *DRB1-DPB1* haplotype | D’ | MS | HCs |  |  |
| 0101-0402 | 0.72 | 0.037 | 0.058 | 0.1838 | 1 |
| 0101-0501 | 0.56 | 0.009 | 0.015 | 0.4268 | 1 |
| 0403-0201 | 0.31 | 0.014 | 0.011 | 0.7322 | 1 |
| 0403-0501 | 0.29 | 0.016 | 0.016 | 0.9826 | 1 |
| 0405-0201 | 0.23 | 0.043 | 0.017 | 0.0156 | 0.3276 |
| 0405-0301 | 0.39 | 0.045 | 0.009 | 0.0002 | 0.0042 |
| 0405-0402 | 0.04 | 0.033 | 0.015 | 0.0705 | 1 |
| 0405-0501 | 0.23 | 0.121 | 0.091 | 0.1455 | 1 |
| 0406-0201 | 0.23 | 0.023 | 0.013 | 0.2742 | 1 |
| 0406-0501 | 0.07 | 0.032 | 0.012 | 0.0268 | 0.5628 |
| 0802-0501 | 0.10 | 0.021 | 0.019 | 0.8347 | 1 |
| 0803-0202 | 0.52 | 0.020 | 0.013 | 0.4293 | 1 |
| 0803-0501 | 0.27 | 0.042 | 0.048 | 0.7017 | 1 |
| 0901-0201 | 0.11 | 0.010 | 0.042 | 0.0099 | 0.2079 |
| 0901-0501 | 0.10 | 0.029 | 0.072 | 0.0100 | 0.2100 |
| 0901-0901 | 0.01 | 0.007 | 0.016 | 0.2590 | 1 |
| 1201-0201 | 0.16 | 0.014 | 0.014 | 0.9831 | 1 |
| 1201-0501 | 0.22 | 0.016 | 0.015 | 0.8830 | 1 |
| 1501-0201 | 0.13 | 0.048 | 0.022 | 0.0237 | 0.4977 |
| 1501-0501 | 0.15 | 0.078 | 0.042 | 0.0189 | 0.3969 |
| 1501-0901 | 0.83 | 0.065 | 0.098 | 0.0994 | 1 |

p^uncorr^ was corrected by multiplying the value by 21 to calculate p^corr^.

HCs, healthy controls; MS, multiple sclerosis.
