# Supplementary material for: Genetic and Infectious Profiles of Japanese Multiple Sclerosis Patients
Source: PLoS One. 2012 Nov 9;7(11):e48592. doi: 10.1371/journal.pone.0048592 (PMC3494689; doi:10.1371/journal.pone.0048592)
Supplement: Table S4 — Demographic features of MS patients without LESCLs according to the presence or absence of the HLA-DRB1*0405 allele. Exclusion of eight MS patients with LESCLs gave essentially the same results; MS patients positive for DRB1*0405 showed a significantly earlier age of onset and a significantly lower PI compared with those without this allele. (DOC) [file pone.0048592.s004.doc]

**Supplementary Table 4.** Comparison of demographic features and clinical characteristic of MS patients without LESCLs according to the presence or absence of the *HLA-DRB1*0405* allele

|  | *0405* (+) (n = 62) | *0405* (-) (n = 75) | p^uncorr^ | p^corr^ |
| --- | --- | --- | --- | --- |
| Male:female | 22 : 40 | 25 : 50 | 0.8573 | 1 |
| Age at onset (years)^a^ | 27.00 ± 10.67 | 35.00 ± 13.93 | 0.0012 | 0.0096 |
| Disease duration (years)^a^ | 12.82 ± 9.88 | 10.15 ± 7.41 | 0.1242 | 0.9936 |
| EDSS score^a^ | 2.41 ± 1.97 | 3.39 ± 2.26 | 0.0080 | 0.0640 |
| Annualized relapse rate^a^ | 0.53 ± 0.44 | 0.71 ± 0.80 | 0.2505 | 1 |
| Progression Index^a^ | 0.32 ± 0.42 | 0.65 ± 1.41 | 0.0013 | 0.0104 |
| OB/increased IgG index^b^ | 22/40 (52.5%) | 35/50 (70.0%) | 0.1255 | 1 |
| Barkhof criteria^c^ | 28/55 (50.9%) | 47/65 (72.3%) | 0.0228 | 0.1824 |
| ^a^Values represent the mean ± SD.  ^b^CSF oligoclonal IgG bands (OB) and/or increased IgG index (upper normal limit = 0.658, according to our previous study [21].  ^c^Brain MRI lesions that meet the Barkhof criteria [29].  EDSS, Kurtzke’s Expanded Disability Status Scale; LESCLs, longitudinally extensive spinal cord lesions extending over three or more vertebral segments; MS, multiple sclerosis; OB, oligoclonal IgG bands.  p^uncorr^ was corrected by multiplying the value by eight to calculate p^corr^. | | | | |
